# Four cases of completion lobectomy for locally relapsed lung cancer after segmentectomy

**DOI:** 10.1186/s12957-021-02165-x

**Published:** 2021-02-11

**Authors:** Shigeki Suzuki, Keisuke Asakura, Kyohei Masai, Kaoru Kaseda, Tomoyuki Hishida, Hisao Asamura

**Affiliations:** Division of Thoracic Surgery, Keio University School of Medicine, 35, Shinanomachi, Shinjuku-ku, Tokyo, 160-8582 Japan

**Keywords:** Completion lobectomy, Local recurrence, Lung cancer, Post segmentectomy

## Abstract

**Background:**

Although completion lobectomy is the treatment of choice for local recurrence of non-small cell lung cancer after segmentectomy, few cases have been reported. We report four patients who underwent completion lobectomies for staple line recurrence after segmentectomy for stage I non-small cell lung cancer.

**Case presentation:**

Three women aged 65, 82, and 81 years underwent completion lower lobectomy after superior segmentectomy of the same lobe for local recurrence of stage I non-small cell lung cancer. A 67-year-old man, who had a tumor recurrence on the staple line after apical segmentectomy with superior mediastinal nodal dissection for stage I non-small cell lung cancer, underwent completion right upper lobectomy. These four patients underwent segmentectomy because of comorbidities or advanced age. Local recurrence was confirmed by computed tomography-guided needle biopsy. The interval between the two operations was 37, 39, 41, and 16 months, respectively. Although minimal hilar adhesion was seen for the three completion lower lobectomies, tight adhesions after apical segmentectomy made completion right upper lobectomy quite difficult to dissect, which led to injury of the superior pulmonary vein. No recurrence was recorded after completion lobectomies for 62, 70, 67, and 72 months, respectively.

**Conclusions:**

Although completion lobectomy is one of the most difficult modes of resection, among several completion lobectomies, completion lower lobectomy after superior segmentectomy without superior mediastinal nodal dissection was relatively easy to perform because of fewer hilar adhesions.

**Supplementary Information:**

The online version contains supplementary material available at 10.1186/s12957-021-02165-x.

## Background

Although the current gold standard for the surgical treatment of lung cancer is lobectomy, some retrospective studies have reported that the prognosis after segmentectomy is not inferior to that after lobectomy in patients with stage IA non-small cell lung cancer (NSCLC) [1–3]. However, local recurrence in the remaining lobe after segmentectomy, especially on the staple line, has become a subject for discussion. In this report, we refer to local recurrence on the staple line as “staple line recurrence.” Some studies have shown a local recurrence rate of 4.9 to 5.5% after segmentectomy in patients with stage IA NSCLC [1–3]. Although completion lobectomy is the treatment of choice for staple line recurrence of NSCLC after segmentectomy, few cases have been reported [4, 5]. We report four patients who underwent completion lobectomy for local relapsed NSCLC after segmentectomy and discuss the differences in technical difficulties among them.

## Case presentation

Between 2007 and 2011, 532 consecutive patients with NSCLC underwent complete tumor resection in our institution. These included 406 (81.9%) patients with clinical stage I NSCLC, among whom 121 (29.8%) underwent segmentectomy. Seven (5.8%) of these patients developed local recurrence in the same lobe. Four of these underwent completion lobectomy for staple line recurrence. The other three patients underwent photodynamic therapy for recurrence on the bronchial stump, stereotactic radiation because of advanced age, and best supportive care because of advanced age. In the four patients who underwent completion lobectomy, although their predicted postoperative pulmonary function showed that they could tolerate lobectomy at the timing of initial surgery, we performed segmentectomy because of their comorbidities or advanced age.

### Patient 1

**Fig. 1:**
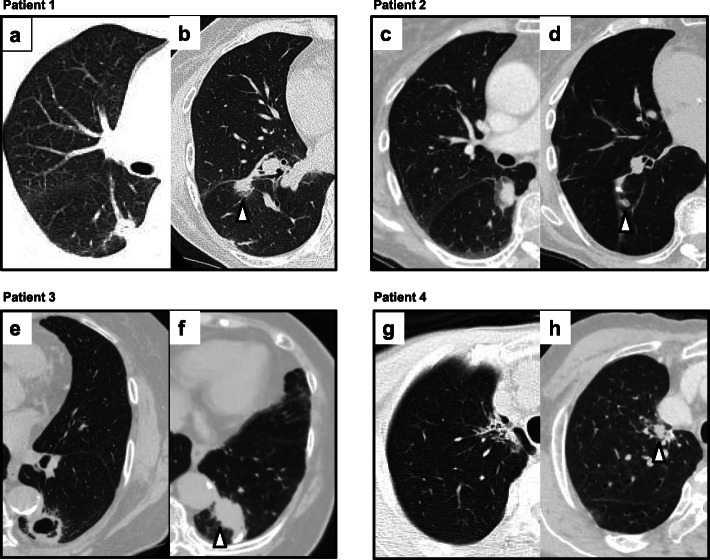
CT findings in the four patients. **a** Primary lesion in the right superior segment in patient 1; 15 mm in diameter. **b** Recurrent lesion (37 months after segmentectomy) on the staple line in patient 1 (arrowhead); 20 mm. **c** Primary lesion in the right superior segment in patient 2 (27 mm). **d** Recurrent lesion (39 months after segmentectomy) on the staple line in patient 2 (arrowhead) (10 mm). **e** Primary lesion in the left superior segment in patient 3 (28 mm). **f** Recurrent lesion (41 months after segmentectomy) on the staple line in patient 3 (arrowhead) (45 mm). **g** Primary lesion in the right apical segment in patient 4 (35 mm). **h** Recurrent lesion (16 months after segmentectomy) on the staple line in patient 4 (arrowhead) (10 mm)

Patient 1 was a 65-year-old woman who underwent right superior segmentectomy for c-T1aN0M0 adenocarcinoma (diameter, 15 mm) because of coexistent advanced vulvar cancer. Thirty-seven months after segmentectomy, chest computed tomography (CT) indicated a 20-mm nodule adjacent to the staple line in the periphery of the remaining lobe (Fig. 1a, b). CT-guided needle biopsy (CTNB) revealed evidence of local recurrence. At the time of recurrence, the vulvar cancer had been judged to be stable, and thus a completion right lower lobectomy was performed. There were minimal hilar and interlobar adhesions in the completion lobectomy, resulting in successful completion without any vessel injuries. The operation time was 167 min and the blood loss was 75 ml. She was discharged on postoperative day 9 without any complications. She died of endometrial cancer 62 months after completion lobectomy without recurrence of lung cancer.

### Patient 2

Patient 2 was an 82-year-old woman who underwent right superior segmentectomy for c-T1bN0M0 adenocarcinoma (diameter, 27 mm) because of her advanced age. Thirty-nine months after segmentectomy, chest CT indicated a 10-mm nodule adjacent to the staple line in the periphery of the remaining lobe (Fig. 1c, d). Local recurrence was revealed by CTNB. A completion right lower lobectomy was performed for the recurrence, in accordance with the patient’s preference. There were minimal hilar and interlobar adhesions in the completion lower lobectomy, resulting in successful completion without any vessel injuries. The operation time was 255 min and the blood loss was 220 ml. She was discharged on postoperative day 7 without any complications. To date, she has survived disease-free for 70 months after completion lobectomy.

### Patient 3

Patient 3 was an 81-year-old woman who underwent left superior segmentectomy for c-T1bN0M0 squamous cell carcinoma (diameter, 28 mm) in view of her advanced age. Forty-one months after segmentectomy, chest CT indicated a 45-mm mass adjacent to the staple line in the periphery of the remaining lobe (Fig. 1e, f). Local recurrence was revealed by CTNB. At the time of recurrence, a completion left lower lobectomy was performed based on her preference, despite her advanced age. There were minimal hilar adhesions in completion lower lobectomy. Although there were minor adhesions around the bronchial stump of the prior segmentectomy, we successfully performed completion lower lobectomy without any vessel injuries. The operation time was 316 min and the blood loss was 100 ml. She was discharged on postoperative day 18 without any complications. To date, she has survived disease-free for 67 months after completion lobectomy.

### Patient 4

Patient 4 was a 67-year-old man who underwent right apical segmentectomy and superior mediastinal nodal dissection for c-T2aN0M0 adenocarcinoma (diameter, 35 mm) because of chronic obstructive pulmonary disease plus renal failure. Sixteen months after segmentectomy, chest CT indicated a 10-mm nodule adjacent to the staple line in the center of the remaining lobe (Fig. 1g, h). Local recurrence was revealed by CTNB. At the time of recurrence, he opted for completion right upper lobectomy despite his requirement for dialysis. There were tight adhesions around the hilum due to the prior segmentectomy and superior mediastinal nodal dissection. The superior pulmonary vein injury occurred when we dissected it at the hilum. To control the bleeding, we clamped the right main pulmonary artery and superior pulmonary vein through the pericardium because we could not dissect and encircle the tape around them at the hilum. After that, we divided the right upper lobe pulmonary vein. The operation time was 284 min and the blood loss was 370 ml. He was discharged on postoperative day 21 with a phrenic nerve palsy, from which he completely recovered within 3 months. To date, he has survived disease-free for 72 months after completion lobectomy.

## Discussion and conclusions

We reported four patients who underwent completion lobectomy for staple line recurrence after segmentectomy as limited resection for stage I NSCLC. Three of these four patients underwent completion lower lobectomy after superior segmentectomy without any vessel injury. In one patient who underwent completion right upper lobectomy after apical segmentectomy, we accidentally injured the superior pulmonary vein due to tight hilar adhesions, and intrapericardial vessel clamps were required to control the bleeding. There are at least three possible explanations for the differences in technical difficulty between completion upper lobectomy after apical segmentectomy and completion lower lobectomy after superior segmentectomy.

The first explanation is the anatomical feature of the superior segment, in which all the bronchovascular structures (pulmonary artery, vein, and bronchus) independently arise from those of the basal segments. This feature is only seen in the superior segment. Therefore, there are minimal adhesions around the remaining bronchovascular structures of the basal segments after superior segmentectomy, and thus, dissection was relatively easy in completion lower lobectomy. On the other hand, in the upper lobe, bronchovascular structures of the respective segments (apical, posterior, and anterior segment) are adjacent to each other. This resulted in postoperative adhesions around the vessels/bronchi of the other segments of the upper lobe after segmentectomy.

The second explanation is the presence of adhesions around the main pulmonary artery at the hilum. In the case of completion upper lobectomy after apical segmentectomy, there were tight adhesions around the hilum and this prevented us from securing the main pulmonary artery at the hilum. Therefore, we had to perform intrapericardial vessel clamp to control bleeding. In upper lobe segmentectomy, dissection around the main pulmonary artery at the hilum is performed, resulting in the formation of adhesions around the hilum. The tight hilar adhesions after segmentectomy of the upper lobe made the completion lobectomy quite difficult. In contrast, in lower lobe segmentectomy, dissection around the main pulmonary artery at the hilum is not necessarily performed, resulting in the formation of minimal adhesions around the hilum. Thus, at the time of completion lower lobectomy, in most cases, there are no restrictions. In their summary of 10 completion lobectomies (six completion upper lobectomies and four completion lower lobectomies), Takahashi et al. reported that there were tight hilar adhesions in only one out of four completion lower lobectomies (25%). This was in contrast to the presence of hilar adhesions in all six completion upper lobectomies [5].

A third possible explanation is the effect of superior mediastinal nodal dissection carried out during segmentectomy on the formation of hilar adhesions. The anatomical landmark for the proximal end of the superior mediastinal nodal dissection is the upper limb of the pulmonary artery at the hilum, which is why there are tight hilar adhesions. In one of our patients who underwent completion right upper lobectomy after apical segmentectomy and superior mediastinal nodal dissection, we injured the superior pulmonary vein inadvertently due to the hilar adhesions. Takahashi et al. reported that pulmonary artery injury occurred in two of five (40%) completion lobectomies after upper lobe segmentectomy and superior mediastinal nodal dissection [5]. Completion upper lobectomy is technically more difficult than completion lower lobectomy because of the three reasons described above. Moreover, the impact on postoperative pulmonary function after right upper lobectomy is less than that after lower lobectomy, because right upper lobe has lower lung volume than lower lobe. Therefore, we should consider the indication of segmentectomy carefully for the tumor in right upper lobe than that in other lobes.

Table S1 summarizes previously reported cases of completion lobectomy after segmentectomy for pulmonary malignancy [4, 5]. We excluded reports of completion lobectomy after wedge resection [6] or after segmentectomy due to a short surgical margin [7] or unanticipated lymph node metastasis [8]. Omasa et al. reported 11 patients (9 primary lung cancer, 2 metastatic lung cancer) who underwent completion lobectomy due to perioperative complication (*n* = 3), unanticipated lymph node metastasis (*n* = 3), or appearance of malignancy (2 local recurrence and 3 new malignancy) [4]. They concluded that completion lobectomy may become more difficult approximately 5 weeks after prior segmentectomy, due to tight adhesion, but can be performed safely with careful manipulation such as securing the main pulmonary artery. Although they did not provide details regarding the patients and respective perioperative data, 5 of their 11 patients underwent completion lobectomy after segmentectomy due to the appearance of malignancy. Takahashi et al. reported 10 patients (7 primary lung cancer, 3 metastatic lung cancer) who underwent completion lobectomy due to the appearance of malignancy (4 local recurrence, 6 new malignancy) [5]. They reported a patient who underwent completion lower lobectomy after superior segmentectomy, where there was no tight hilar adhesion, and showed that it can be performed without intraoperative vessel injury, as in our 3 patients who underwent completion lower lobectomy after superior segmentectomy. To the best of our knowledge, this is the first case series reporting long-term prognosis after completion lobectomy for locally relapsed NSCLC after radical segmentectomy. In our series, with a median follow-up time of 68.5 months (62–72 months) after completion lobectomy, there was no recurrence or death due to lung cancer.

In conclusion, although completion lobectomy is one of the most difficult mode of resection, among several completion lobectomies, completion lower lobectomy after superior segmentectomy without superior mediastinal nodal dissection was relatively easy to perform because there were fewer hilar adhesions.

## Supplementary Information

**Additional file 1: Table S1.** Summary of previous reports of completion lobectomy long after segmentectomy for pulmonary malignancy.

## Data Availability

Not applicable.

## Abbreviations

CT: Computed tomography
CTNB: Computed tomography-guided needle biopsy
NSCLC: Non-small cell lung cancer

## References

[1] Keenan RJ, Landreneau RJ, Maley RH, Singh D, Macherey R, Bartley S (2004). Segmental resection spares pulmonary function in patients with stage I lung cancer. Ann Thorac Surg..

[2] Okada M, Koike T, Higashiyama M, Yamato Y, Kodama K, Tsubota N (2006). Radical sublobar resection for small-sized non-small cell lung cancer: a multicenter study. J Thorac Cardiovasc Surg..

[3] Landreneau RJ, Normolle DP, Christie NA, Awais O, Wizorek JJ, Abbas G (2014). Recurrence and survival outcomes after anatomic segmentectomy versus lobectomy for clinical stage I non-small-cell lung cancer: a propensity-matched analysis. J Clin Oncol..

[4] Omasa M, Date H, Takamochi K, Suzuki K, Miyata Y, Okada M (2016). Completion lobectomy after radical segmentectomy for pulmonary malignancies. Asian Cardiovasc Thorac Ann..

[5] Takahashi Y, Miyajima M, Tada M, Maki R, Mishina T, Watanabe A (2019). Outcomes of completion lobectomy long after segmentectomy. J Cardiothorac Surg..

[6] Wang Y, Wang R, Zheng D, Han B, Zhang J, Zhao H (2017). The indication of completion lobectomy for lung adenocarcinoma </=3 cm after wedge resection during surgical operation. J Cancer Res Clin Oncol..

[7] Liu YW, Chou SH, Hung JY, Kao CN, Chang PC (2019). Thoracoscopic completion right lower lobectomy after anteromedial basilar segmentectomy in early-stage lung cancer. Thoracic cancer..

[8] Nomori H, Mori T, Izumi Y, Kohno M, Yoshimoto K, Suzuki M (2012). Is completion lobectomy merited for unanticipated nodal metastases after radical segmentectomy for cT1 N0 M0/pN1-2 non-small cell lung cancer?. J Thorac Cardiovasc Surg..

